# Quantitative refinement and multicentre psychometric validation of the Chinese total knee arthroplasty patient-reported outcome measure: a prospective cohort study with repeated measures

**DOI:** 10.1016/j.lanwpc.2026.101989

**Published:** 2026-09-19

**Authors:** Chao Xu, Xiaofeng Chang, Feng Ye, Haodong Wu, Guo Chen, Rui Zhang, Shuxin Yao, Lei Shang, Jianbing Ma

**Affiliations:** a Department of Knee Joint Surgery, Honghui Hospital, Xi'an Jiaotong University, No.555 E. Youyi Road, Xi'an, Shaanxi, China; b Faculty of Preventive Medicine, Department of Health Statistics, Fourth Military Medical University, No.169 W. Changle Road, Xi'an, Shaanxi, China; c Department of Neurobiology, School of Basic Medicine, Fourth Military Medical University, Xi'an, 710032, China; d Department of Hip and Knee Arthropathies, Sichuan Provincial Orthopedic Hospital, No.132 West First Section First Ring Road, Chengdu, Sichuan, China; e Department of Joint and Bone 1, Gansu Provincial Hospital of Traditional Chinese Medicine, 418 Guazhou Road, Lanzhou, Gansu, China

**Keywords:** Total knee arthroplasty (TKA), Patient-reported outcome measure (PROM), Prospective cohort study, Psychometric validation, China, Chinese TKA patient reported outcome measure (CTP)

## Abstract

**Background:**

Total knee arthroplasty (TKA) requires valid, culturally appropriate patient-reported outcome measures (PROMs). A 35-item preliminary Chinese Total Knee Arthroplasty Patient-reported Outcome Measurement (CTP) was previously developed by our group through qualitative research with Chinese patients and multidisciplinary experts, but it had not undergone quantitative refinement or prospective multicentre psychometric validation. The present study aimed to refine the instrument and evaluate the psychometric properties of its operated-knee core score.

**Methods:**

We conducted a prospective multicentre cohort psychometric study with repeated assessments at tertiary hospitals in Xi'an, Chengdu, and Lanzhou. Participants were recruited from June 2023 to June 2024, with 12-month follow-up continuing through June 2025. Three independent cohorts were used for quantitative item screening, final scale construction, and psychometric evaluation. We assessed internal consistency (Cronbach's α), test–retest reliability (ICC), structural validity, convergent validity (correlations with WOMAC), and responsiveness (effect size [ES]; standardised response mean [SRM]).

**Findings:**

The three analytic cohorts included 321, 529, and 898 participants, respectively (total n = 1748). Across the three cohorts, the mean age was 66·5 years (SD 7·2), and 1195 participants (68·4%) were female. Exploratory factor analysis supported a three-factor structure (Function, Symptoms, Quality of Life) explaining 72·2% of variance, with all item loadings ≥0·40. The final clinical questionnaire contained 22 items. The principal psychometric analyses evaluated a 16-item operated-knee core score, which showed high internal consistency (Cronbach's α = 0·864) and good test–retest reliability (ICC = 0·836). Convergent validity was supported by a strong correlation between CTP Function and WOMAC Function (r = 0·698; 95% CI 0·663–0·730; p < 0·001). At 12 months, the mean operated-knee core score decreased from 33·52 (SD 8·74) to 21·81 (SD 9·81), with an ES of 1·34 and an SRM of 1·66.

**Interpretation:**

The final CTP is a 22-item, target-population-informed clinical PROM. Its 16-item operated-knee core score showed satisfactory reliability and responsiveness, with partial support for the proposed three-factor structure and convergent validity for selected overlapping constructs.

**Funding:**

National Natural Science Foundation of China, Key Research and Development Program of Shaanxi Province, Cultivation Project for General Project of Xi'an Health Commission, Xi'an Municipal Science and Technology Plan Project: General Medical Research Project, the Program for Innovation Team of Shaanxi Province.

**Research in context**

**Evidence before this study**

We searched PubMed, Embase, Scopus, CNKI, Wanfang Data Knowledge Service Platform, and VIP Chinese Journal Service Platform from database inception to May 2026 for studies of patient-reported outcome measures (PROMs) in total knee arthroplasty (TKA). Search terms covered three main concepts: PROMs, measurement properties or psychometric evaluation, and knee arthroplasty or replacement. We included full-text studies published in English or Chinese that reported the development, cultural adaptation, or evaluation of measurement properties of PROMs used in adults undergoing, awaiting, or followed after TKA. We excluded studies that did not evaluate measurement properties, merely used established PROMs as study outcomes, did not focus on TKA populations, evaluated non-patient-reported measures, or were not available as full reports. Studies relevant to Chinese patients were then examined to determine whether any multidomain, TKA-specific PROM had been developed de novo with Chinese patients and prospectively validated. Existing studies predominantly concerned translated or cross-culturally adapted instruments. To our knowledge, we identified no previous de novo, multidomain TKA-specific PROM developed with Chinese patients that had undergone prospective multicentre psychometric validation. Full database-specific search strategies and eligibility criteria are provided in Supplementary Methods S1.

**Added value of this study**

To our knowledge, this study provides the first prospective multicentre psychometric validation of a de novo, multidomain TKA-specific PROM developed with and for Chinese patients. Building on our previously reported qualitative development work, including patient interviews and Delphi expert review, the present study used three independent multicentre cohorts for quantitative item refinement and prospective psychometric evaluation. The 16-item operated-knee core score demonstrated satisfactory reliability and responsiveness, with partial support for the proposed three-factor structure and convergent validity for selected overlapping constructs. It includes activities and social contexts relevant to the target population, such as squatting, rising from low seating, mild field labour, and community exercise.

**Implications of all the available evidence**

The CTP provides a standardised, culturally grounded approach to assessing TKA outcomes in Chinese patients and may support clinical research and patient-centred care. Future studies should establish interpretability thresholds and validate the instrument in more geographically diverse populations.

## Introduction

With the growing emphasis on patient-centred care, the evaluation of treatment and rehabilitation outcomes increasingly prioritises patients' perspectives over clinician-based assessments.^1^ This paradigm shift acknowledges that patients' experiences, values, and goals are essential to fully understanding the impact of healthcare interventions. Patient-Reported Outcome Measures (PROMs) provide direct insight into aspects that matter most to patients, such as quality of life, emotional well-being, and functional status.^2^ Compared to traditional clinical indicators, PROMs offer several advantages: (1) Enhanced communication and trust between patients and clinicians^3^; (2) Early identification of issues and individualised care; (3) Comprehensive assessment encompassing physical, emotional, and social health^4^; (4) More accurate detection of meaningful improvements from the patient's perspective; (5) Reduced biases in physician evaluations^5^; and (6) the ability to monitor patients remotely and adjust interventions in a timely manner.^6^

Knee osteoarthritis (KOA), the most prevalent degenerative joint disease, significantly impairs patients’ quality of life. In China, the pooled prevalence of symptomatic KOA is approximately 14·6%.^7^ Total knee arthroplasty (TKA) remains the standard treatment for end-stage KOA, from 2012 to 2019, TKA procedures in China increased at an annual rate of 27·43%,^8^ yet 10–20% of patients report dissatisfaction after surgery,^9^ highlighting the growing need for effective evaluation tools to guide postoperative care and improve outcomes.

Several instruments are currently used to assess TKA outcomes, including the Oxford Knee Score (OKS), the new Knee Society Score (new-KSS), and the Western Ontario and McMaster Universities Osteoarthritis Index (WOMAC). However, assessments based on the Consensus-based Standards for the Selection of Health Measurement Instruments (COSMIN)^10^ have identified methodological limitations in these tools.^11^^,^^12^ For example, the OKS was rated as inadequately developed due to non-representative sampling and unclear construct definitions,^13^ while the new-KSS lacked sufficient patient involvement and robust validation, leading to moderate to low-quality evidence for key psychometric properties such as internal consistency and construct validity.^12^ Furthermore, ceiling effects observed in the Knee Injury and Osteoarthritis Outcome Score (KOOS) limit their sensitivity in detecting postoperative improvements.^12^ Pain, stiffness, and basic limitations in mobility are not culturally exclusive constructs, and established PROMs remain valuable after appropriate translation and validation. However, content validity also depends on whether the activities and social contexts represented by an instrument are relevant to the target population. Our previous qualitative work identified several activities and social contexts of particular relevance to Chinese patients undergoing TKA, including squatting and other deep–flexion activities, rising from low seating without arm support, mild field labour, community exercise such as Tai Chi or square dancing, and the impact of knee symptoms on visiting friends and relatives.^14^

To date, most clinical assessments rely on translated versions of Western instruments, which may not adequately reflect the cultural, linguistic, and socio-economic context of Chinese patients.^15^^,^^16^ Our group previously developed a 35-item preliminary CTP through qualitative work with Chinese patients and multidisciplinary experts, but that preliminary instrument had not undergone quantitative refinement or prospective multicentre psychometric validation. This study aimed to quantitatively refine a culturally appropriate PROM—the Chinese Total Knee Arthroplasty Patient-reported Outcome Measurement (CTP)—to assess the effectiveness of TKA in Chinese patients. The rationale for the CTP is therefore to complement established instruments with target-population-informed content rather than to replace established PROMs. Additionally, we evaluated the CTP's reliability, structural validity, convergent validity, and responsiveness. This study provides a standardised tool for outcome assessment in Chinese TKA patients, supporting improved clinical decision-making, postoperative management, and resource allocation.

## Methods

### Study design and participants

This prospective multicentre cohort psychometric study with repeated assessments recruited participants from June 2023 to June 2024 at tertiary hospitals in Xi'an, Chengdu, and Lanzhou, China; 12-month follow-up was completed by June 2025. A flowchart of study procedures is shown in Fig. 1. This prospective cohort study was reported in accordance with the STROBE (Strengthening the Reporting of Observational Studies in Epidemiology) statement.^17^

**Fig. 1:**
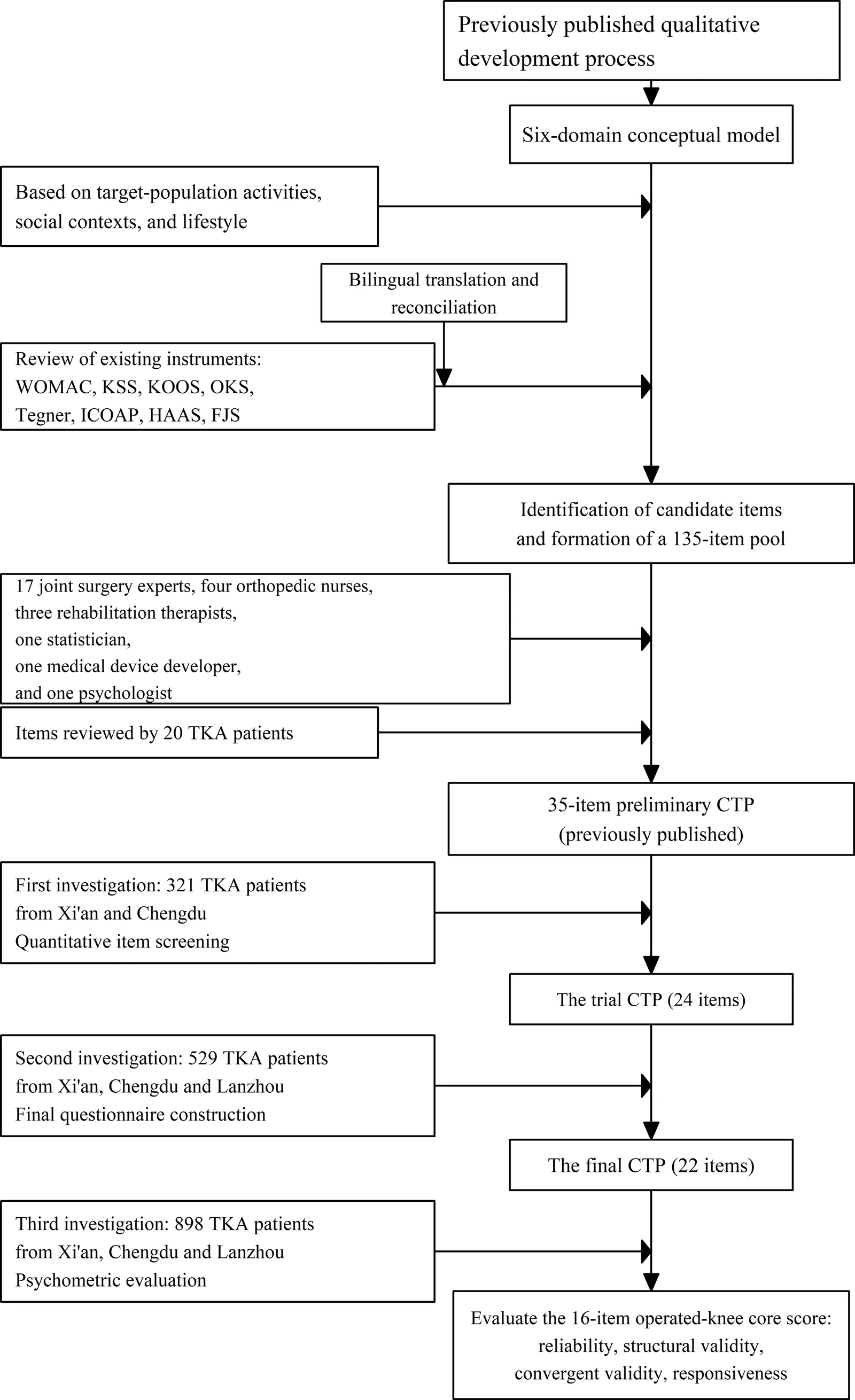
**Overview of the development, quantitative refinement, and psychometric evaluation of the Chinese total knee arthroplasty patient-reported outcome measure.** The qualitative development process and 35-item preliminary CTP were reported previously. Three sequential, independent cohorts were used in the present study for quantitative item screening, final questionnaire construction, and psychometric evaluation. The final clinical CTP contains 22 items, while the principal psychometric analyses evaluated the 16-item operated-knee core score.

Patients scheduled for primary unilateral TKA for end-stage knee osteoarthritis (KOA) were screened in outpatient clinics. After confirming eligibility per predefined criteria, trained staff explained the study and administered paper questionnaires on-site; responses were checked immediately and missing items followed up. Data were collected anonymously. Biological sex was recorded as male or female based on electronic medical record. Gender identity was not collected because it was not included in the prespecified data-collection protocol. Race and ethnicity were not collected because these variables were not included in the original psychometric dataset.

Inclusion criteria were age ≥50 years, confirmed unilateral end-stage KOA, and scheduled primary TKA within six months.^18^^,^^19^ Exclusion criteria included comorbidities affecting lower-limb function or causing pain (eg, stroke, fractures, ligament injuries, vascular or nerve disorders), prior lower-limb revision surgery, and communication impairments preventing questionnaire completion.

### Study phases

The quantitative refinement and validation study comprised three sequential and independent cohorts. Participants did not overlap across phases; no patient contributed data to more than one study cohort.

First phase (quantitative item screening): participants were recruited from the Xi'an and Chengdu centres and completed the previously published 35-item preliminary CTP. This cohort was used to evaluate item performance and conduct the first round of quantitative item reduction, resulting in the 26-item trial CTP. The three Satisfaction items were then consolidated into one global postoperative Satisfaction item, producing the 24-item trial CTP administered in Sample 2.

Second phase (final scale construction): a separate cohort recruited across the Xi'an, Chengdu, and Lanzhou centres completed the 24-item trial CTP. Further quantitative item refinement and expert review resulted in the final 22-item clinical CTP.

Third phase (psychometric evaluation): a third independent cohort recruited across the Xi'an, Chengdu, and Lanzhou centres completed the final 22-item CTP. The operated-knee core scoring set was used for the principal psychometric analyses.

### Assessment timing

At baseline, the 16-item operated-knee core CTP and WOMAC were administered after enrolment and before TKA. The global postoperative Satisfaction item was not assessed at baseline. For responsiveness, the 16-item operated-knee core CTP and WOMAC were repeated at 12 months after TKA (allowable window ±1 month), and the global Satisfaction item was assessed separately as a postoperative appraisal. For test–retest reliability, A computer-generated random sample of 150 participants was selected from the pooled Sample 3 cohort across the three study centres. Participants completed the CTP twice preoperatively at a 2-week interval, with no surgical or other relevant clinical intervention between assessments. Baseline demographic characteristics of the retest subgroup were compared with those of the remaining Sample 3 participants who did not undergo retest assessment. Standardised mean differences were used as the primary measure of baseline imbalance, supplemented by Welch t tests for continuous variables and Pearson chi-square tests for categorical variables.

### Previous conceptual development of the preliminary CTP

The conceptual model and 35-item preliminary CTP were developed and reported previously. Briefly, the previous study constructed a conceptual model pool from a targeted review of TKA PROMs and relevant content from the China Health and Retirement Longitudinal Study, followed by patient and multidisciplinary-expert concept elicitation, two rounds of Delphi consultation, and cognitive debriefing. Combined with a forward-backward translation procedure and adaptation to Chinese cultural characteristics, an initial pool of 135 items was identified. This 135-item pool underwent a systematic screening process involving evaluation by a multidisciplinary panel (17 joint surgery experts, four orthopedic nurses, three rehabilitation therapists, two medical statisticians, one medical device developer, and one psychologist) and cognitive debriefing with 20 TKA patients. Through these two rounds of evaluation, the item pool was reduced from 135 to 35 items. This process produced a six-domain, 35-item preliminary CTP. The present study did not repeat that qualitative development process; instead, it began with the published 35-item preliminary instrument and focused on quantitative item refinement and psychometric validation. A complete audit trail of the 35 original items, including their Chinese and English wording, provenance, stage-specific disposition, and rationale for retention, removal, or consolidation, is provided in Supplementary Table S1A and S1B.

### Scoring

The final clinical questionnaire contains 22 items: 10 bilateral symptom items (five for each knee), eight Function items, three Quality-of-Life items, and one global postoperative Satisfaction item. For psychometric evaluation, the operated-knee core score was based on 16 items: five operated-knee Symptoms items (0–20), eight Function items (0–32), and three Quality-of-Life items (0–12), yielding a core total score of 0–64. The five contralateral-knee symptom items were retained for bilateral clinical assessment but were not included in the operated-knee core score. The global Satisfaction item (0–4) was treated as a separate postoperative appraisal and was not included in the three-factor latent structure, domain internal-consistency analyses, or the core total score.

### Data collection and procedures

Three rounds of data collection were performed by six trained investigators. After consent and questionnaire completion, demographic data were recorded. All completed forms were reviewed on-site and entered into EpiData with double entry and logical checks. Statistical analyses were conducted in SPSS 24·0.

### Sample size

Following psychometric conventions, we targeted 5–10 participants per item plus an estimated 20% attrition, yielding ≥210 participants per round. A G∗Power a priori calculation (Cohen's f = 0·25, α = 0·05, power = 0·95) indicated a minimum of 252 participants, adjusted to ≈315 per round allowing for 20% loss; thus each wave recruited ≥315 patients.

### Quality control and missing data

All questionnaires were reviewed for completeness at the time of administration. When an item was unanswered, the study team attempted to contact the participant to obtain the missing response where feasible. Responses obtained during follow-up were recorded as participant-reported data and were not statistically imputed. No item-level or multiple imputation was performed. If a response required for score calculation or a prespecified psychometric analysis remained unavailable, the questionnaire was excluded from the corresponding analytic dataset. Analyses were therefore conducted using complete cases for the variables required by each analysis. Data were entered independently by two investigators and checked for consistency. Final analytic datasets contained no blank scored-item responses.

### Item analysis and selection

Item-level floor or ceiling concentrations greater than 15% were flagged as one of the prespecified item-performance criteria. Final item-retention decisions were based on the combined quantitative criteria and clinical/content review rather than on any single criterion alone.^18^, ^19^, ^20^ Five quantitative criteria guided item screening: (1) non-significant critical-ratio comparison between the upper and lower 27% groups; (2) SD < 0·85; (3) item–total correlation <0·40; (4) factor loading <0·40; and (5) an increase in Cronbach's α after item deletion. The 0·40 threshold was used to identify items with comparatively weak relationships with the overall scale and was selected as a screening criterion rather than as an automatic deletion rule. Final item-retention decisions were based on the combined quantitative criteria and clinical/content relevance.^21^ Items meeting three or more exclusion criteria were removed; items meeting two criteria underwent clinical and content review.

### Reliability

Internal consistency was assessed by Cronbach's α (acceptable α ≥ 0·70 for total scale, ≥0·60 for subscales) and Guttman split-half reliability. Test–retest reliability was evaluated using intraclass correlation coefficients (ICC).^22^

### Validity and factor analysis

Content validity was appraised by three psychologists and seven orthopedic specialists. The sample was randomly split for EFA and confirmatory factor analysis (CFA) to establish and validate factor structure.^23^ Model fit was evaluated using the standardised root mean square residual (SRMR), the adjusted goodness-of-fit index (AGFI), the goodness-of-fit index (GFI), the comparative fit index (CFI), the non-normed fit index (NNFI), the chi-square/degree-of-freedom ratio (χ^2^/df), and the root mean square error of approximation (RMSEA). Acceptable thresholds were SRMR <0·08, AGFI and GFI >0·90, CFI and NNFI >0·95, χ^2^/df < 5, and RMSEA <0·05.^24^, ^25^, ^26^

### Convergent validity against WOMAC

Convergent validity was examined via Pearson correlations between CTP dimensions and WOMAC; correlation strength was classified as: none (0·00–0·20), weak (0·21–0·40), moderate (0·41–0·60), strong (0·61–0·80), and very strong (0·81–1·00).

### Responsiveness

Responsiveness to change after TKA was evaluated using effect size (ES = mean change/baseline SD) and standardised response mean (SRM = mean change/SD of change scores); both were reported as absolute values (higher = greater improvement). ES/SRM thresholds of 0·2, 0·5, and 0·8 denote small, moderate, and large effects, respectively.^24^, ^25^, ^26^, ^27^ Bootstrap (1000 resamples) was used to estimate 95% CIs for ES and SRM.^28^

### Statistical analysis

Distributional shape was assessed using histograms and Q–Q plots, together with mean–median agreement, skewness, kurtosis, and Shapiro–Wilk tests. Formal normality-test p values were not used as the sole criterion for selecting analytical methods because of the large sample size and the bounded, discrete nature of PROM scores. Approximately symmetric continuous variables are reported as mean (SD), markedly skewed continuous variables as median (IQR), and categorical variables as counts and percentages. Pearson correlations were used for the primary convergent-validity analyses, with Spearman rank correlations conducted as non-parametric robustness analyses. Responsiveness was quantified using effect size and standardised response mean with bootstrap 95% CIs; paired Wilcoxon signed-rank tests were additionally conducted to assess the robustness of within-person changes. All tests were two-sided, and p < 0·05 was considered statistically significant. Exact p values are reported to three decimal places, except for values below 0·001, which are reported as p < 0·001.

### Ethics approval

This study was performed in accordance with the principles of the Declaration of Helsinki. Ethical approval was granted by the Medical Ethics Committee of Honghui Hospital, Xi'an Jiaotong University College of Medicine (No: 202202021, February 25, 2022). Written informed consent was obtained from all participants prior to enrolment.

### Role of the funding source

The funders had no role in study design, data collection, data analysis, data interpretation, writing of the report, or the decision to submit the manuscript for publication.

## Results

The three analytic cohorts included 321, 529, and 898 participants, respectively (total n = 1748). Across the three cohorts, the mean age was 66·5 years (SD 7·2), and 1195 participants (68·4%) were female (Table 1). Phase 1 recruited participants from Xi'an and Chengdu, whereas Phases 2 and 3 recruited participants from Xi'an, Chengdu, and Lanzhou. In Phase 1, 31 of 352 questionnaires (8.8%) did not meet the completeness or quality-control requirements for analysis, leaving 321 valid questionnaires. In Phase 2, 53 of 582 questionnaires (9.1%) were not included, leaving 529 valid questionnaires. In Phase 3, 114 of 1012 questionnaires (11.3%) were not included, leaving 898 valid questionnaires. The three analytic cohorts were independent, with no participant overlap across study phases.

**Table 1:** Demographic characteristics of TKA patients included in the CTP evaluation.

Group	Sample 1 (N = 321)		Sample 2 (N = 529)		Sample 3 (N = 898)	
	N	%	N	%	N	%
Age (Mean ± SD, year)	67·7 ± 7·0		66·1 ± 7·3		66·3 ± 7·2	
<65	98	30·5	211	39·9	349	38·9
65–75	200	62·3	272	51·4	466	51·9
>75	23	7·2	46	8·7	83	9·2
Sex						
Male	96	29·9	170	32·1	287	32·0
Female	225	70·1	359	67·9	611	68·0
Educational level						
Primary school and below	172	53·6	234	44·2	430	47·9
Junior high school	112	34·9	195	36·9	328	36·5
Senior high school or above	37	11·5	100	18·9	140	15·6
Living status						
Living alone	23	7·2	25	4·7	54	6·0
Living with spouse	195	60·7	357	67·5	588	65·5
Living with children	103	32·1	147	27·8	256	28·5
Medical insurance type						
Rural cooperative medical care	227	70·7	402	76·0	668	74·4
Urban residents insurance	24	7·5	32	6·0	67	7·5
Employee medical insurance	70	21·8	95	18·0	163	18·2

TKA: total knee arthroplasty, CTP: Chinese Total Knee Arthroplasty Patient-Reported Outcome Measure.

### Item selection

Sample 1 screening removed nine items from the previously published 35-item preliminary CTP: two pain-location items; three Function items (Jogging, Bath/Shower, and Light household activity); one Quality-of-Life item (Embarrassment when others see the knee); and all three Expectation items. Item-level descriptive statistics and stage-specific dispositions for the original 35-item pool are provided in Supplementary Table S1A and S1B. The three Expectation items showed pronounced ceiling concentrations of 94·7%, 84·7%, and 56·7%, respectively. Jogging also showed a ceiling concentration of 71·7%, whereas Bath/Shower, Light household activity, and Embarrassment when others see the knee showed floor concentrations of 45·8%, 29·3%, and 43·0%, respectively. These distributional findings were considered together with the other prespecified quantitative criteria and clinical/content review.

Removal of the nine items reduced the pool from 35 to 26 items. The three Satisfaction items were subsequently consolidated into one global postoperative Satisfaction item, producing a 24-item trial CTP. In Sample 2, two additional Function items—Bending to floor and Getting in/out of a car—were removed, yielding the final 22-item clinical CTP (Fig. 1).

These modifications yielded the final 22-item clinical CTP, comprising 10 bilateral symptom items, eight Function items, three Quality-of-Life items, and one global Satisfaction item (Supplementary Table S1A and S1B; Supplementary Materials S1).

For Sample 3 psychometric analyses, the five contralateral-knee symptom items were retained for bilateral clinical assessment but excluded from the operated-knee core score, while global Satisfaction was analysed separately. Therefore, the principal psychometric analyses included 16 core items: five Symptoms, eight Function, and three Quality-of-Life items (Table 2).

**Table 2:** Factor loadings of the EFA with promax rotation.

Questions	F1	F2	F3
Pain severity		0·698	
Pain frequency		0·926	
Swelling		0·928	
Stiffness		0·86	
Weakness		0·591	
Walking on a flat surface	0·899		
Climbing up or down stairs	0·946		
Squatting	0·894		
Taking off socks or shoes while sitting	0·909		
Standing up from a low sitting position	0·904		
without using the armrests			
Mild field labour	0·865		
Outdoor fitness activities (Tai Chi or Square dancing)	0·84		
Getting on/off toilet	0·467		
Impact on quality of life			0·793
Impact on visiting friends and relatives			0·857
Anxious or depressed			0·874

EFA: exploratory factor analysis, F1: Function, F2: Symptoms, F3: Quality of life.

The final clinical CTP contains 22 items. Psychometric analyses used the 16-item operated-knee core measure: five operated-knee Symptoms items, eight Function items, and three Quality-of-Life items. Five contralateral-knee symptom items were retained for bilateral clinical description but were excluded from the core latent score. The global postoperative Satisfaction item was analysed separately and was not included in the core total. Getting on/off toilet was assigned to the Function domain.

### Structure of the CTP

Exploratory factor analysis (EFA) on a random half of Sample 3 indicated suitability for factor analysis (Kaiser–Meyer–Olkin = 0·769; Bartlett's test χ^2^ = 6613·784, p < 0·001). The scree plot showed a clear elbow at the fourth factor, with a marked decline in eigenvalues across the first three factors and a substantially flatter slope thereafter, supporting retention of a three-factor solution (Fig. 2). The three factors accounted for 37·389%, 20·860%, and 13·964% of variance, respectively (cumulative 72·213%), and all retained items loaded >0·40 (Table 2). The factors were labelled Function (eight items), Symptoms (five items), and Quality of Life (three items) to reflect their content.

**Fig. 2:**
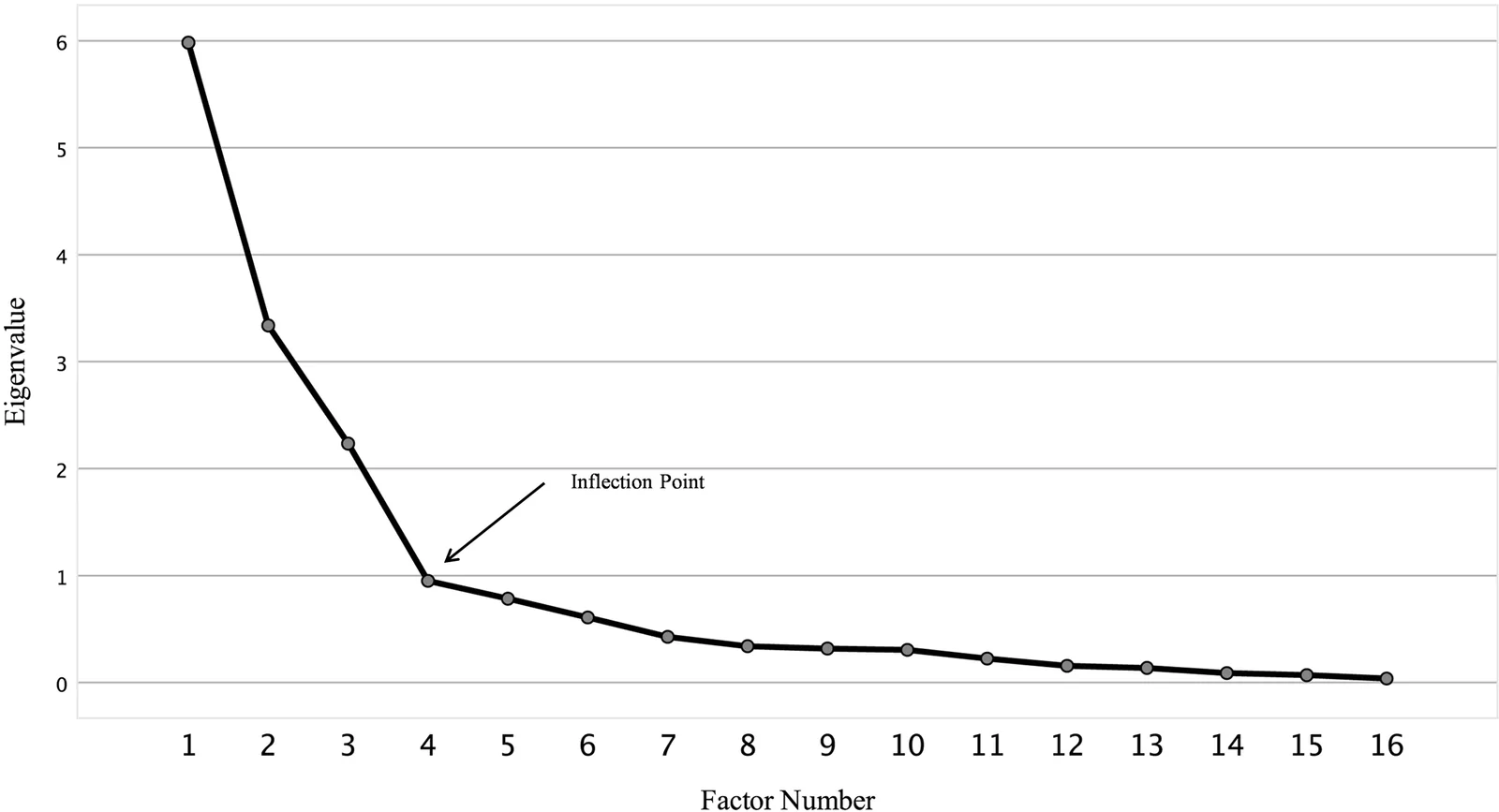
**Scree plot derived from exploratory factor analysis of the CTP items.** The plot displays the eigenvalues of each factor, and the inflection point indicates the optimal number of factors to retain.

### Reliability

Using Sample 3 (n = 898), the 16-item operated-knee core score demonstrated high internal consistency (Cronbach's α = 0·864), with subscale α ranging 0·809–0·942. Guttman split-half reliability for the total scale was 0·782 (subscales 0·790–0·913). Test–retest reliability over two weeks (n = 150) yielded an intraclass correlation coefficient (ICC) of 0·836 for the total scale and 0·811–0·905 for individual dimensions (Table 3), indicating stable temporal reliability.

**Table 3:** Reliability coefficients of the CTP in all dimensions.

Dimensions	Cronbach's α coefficient	Split-half reliability coefficient	Test-retest reliability coefficient
Symptoms	0·867	0·814	0·811
Function	0·942	0·913	0·905
Quality of life	0·809	0·790	0·877
Total	0·864	0·782	0·836

CTP: Chinese Total Knee Arthroplasty Patient-Reported Outcome Measure.

### Validity

Content validity ratio was 0·80. Average completion time was 4·8 ± 1·5 min and participants reported items were easy to understand. Item–total correlations were positive and ranged from 0·544 to 0·906. Confirmatory factor analysis (CFA) on the remaining half of Sample 3 (n = 449) showed mixed model fit. SRMR (0·05), RMSEA (0·04), χ^2^/df (2·13), GFI (0·91), and AGFI (0·90) met the prespecified criteria, whereas CFI (0·92) and NNFI (0·91) were below the prespecified 0·95 threshold. The results therefore provided partial, but not uniformly strong, support for the proposed three-factor structure.^27^

### Convergent validity against WOMAC

CTP Function was strongly correlated with WOMAC Function (r = 0·698; 95% CI 0·663–0·730; p < 0·001). CTP Symptoms was also strongly correlated with WOMAC Pain (r = 0·605; 95% CI 0·562–0·645; p < 0·001). In contrast, CTP Quality of Life showed only a weak correlation with WOMAC Stiffness (r = 0·355; 95% CI 0·296–0·411; p < 0·001), indicating limited overlap between these non-equivalent constructs. These relationships are shown in Fig. 3.

**Fig. 3:**
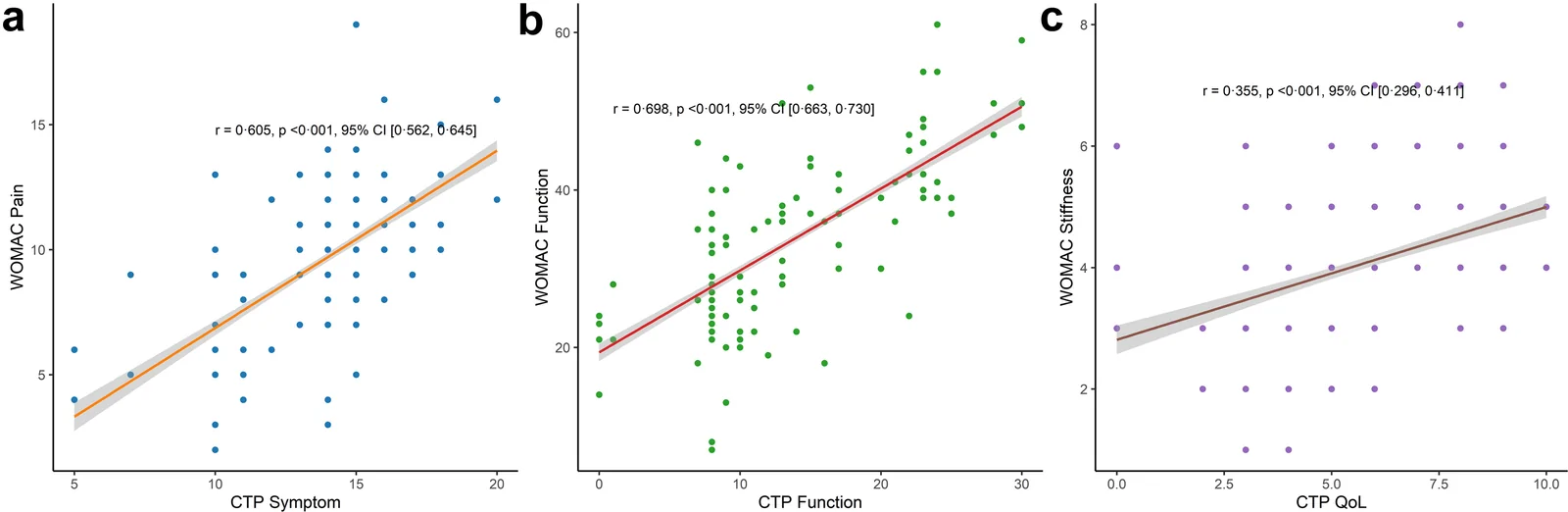
Pearson correlation analyses between corresponding CTP and WOMAC domains in Sample 3. Panels show WOMAC Pain versus CTP Symptom (a), WOMAC Function versus CTP Function (b), and WOMAC Stiffness versus CTP Quality of Life (c). Pearson correlation coefficients, 95% confidence intervals, and p values are displayed.

### Responsiveness

Both the CTP and WOMAC showed large standardised changes from baseline to 1 year after TKA. The CTP total score decreased from 33·52 ± 8·74 at baseline to 21·81 ± 9·81 at 1 year (mean decrease 11·71 ± 7·05; ES = 1·34; SRM = 1·66). CTP subscale ES ranged 0·77–1·12 and SRM 0·82–1·18, indicating moderate-to-large responsiveness. WOMAC total improved from 47·51 ± 12·36 to 27·96 ± 14·15 (mean decrease 19·55 ± 9·87; ES = 1·58; SRM = 1·98), with pain and physical-function subscales showing the largest effects (Pain: ES = 1·41, SRM = 1·70; Function: ES = 1·26, SRM = 1·47). Detailed responsiveness metrics are provided in Table 4.

**Table 4:** Responsiveness indices of CTP and WOMAC scores following total knee arthroplasty (N = 898).

Scales or Dimensions	Baseline		1 year after surgery		Change		ES (95% CI)	SRM (95% CI)
	Mean	SD	Mean	SD	Mean	SD		
CTP								
Symptom	13·80	2·82	10·63	3·93	3·17	2·68	1·12 (1·03–1·22)	1·18 (1·10–1·26)
Function	13·94	7·42	7·18	8·24	6·76	5·83	0·91 (0·84–0·98)	1·16 (1·05–1·26)
QoL	5·78	2·31	4·00	2·73	1·78	2·16	0·77 (0·70–0·84)	0·82 (0·74–0·90)
Total	33·52	8·74	21·81	9·81	11·71	7·05	1·34 (1·26–1·42)	1·66 (1·53–1·79)
WOMAC								
Pain	9·56	3·31	4·89	4·30	4·67	2·74	1·41 (1·31–1·50)	1·70 (1·59–1·81)
Stiffness	4·08	1·42	3·15	2·01	0·93	1·55	0·65 (0·57–0·72)	0·60 (0·52–0·66)
Physical function	33·88	11·06	19·92	12·96	13·96	9·50	1·26 (1·16–1·35)	1·47 (1·37–1·58)
Total	47·51	12·36	27·96	14·15	19·55	9·87	1·58 (1·48–1·68)	1·98 (1·87–2·09)

Data are presented as mean ± standard deviation (SD); Baseline: preoperative score; 1 year after Surgery: score at 1 year postoperative; Change: preoperative minus 1-year postoperative score; positive values indicate improvement (a larger positive value = greater improvement); 95% CI:95% confidence interval (estimated by bootstrap); ES (Effect Size): calculated as change score divided by baseline SD; Positive values indicate improvement. SRM (Standardised Response Mean): calculated as change score divided by SD of the change score.; Positive values indicate improvement; CTP:Chinese Total Knee Arthroplasty Patient-reported Outcome Measurement; WOMAC:Western Ontario and McMaster Universities Osteoarthritis Index; Physical function in WOMAC corresponds to the Function subscale.

Although WOMAC yielded larger ES/SRM for pain and function, the CTP captured substantial change across additional domains (symptoms and psychosocial impact), thus providing complementary information in the Chinese TKA setting.

## Discussion

Building on our previously published conceptual model and 35-item preliminary CTP, the present study quantitatively refined the instrument and evaluated its psychometric properties across three sequential, independent clinical cohorts. The refinement process yielded a final 22-item clinical questionnaire. The principal psychometric analyses focused on a 16-item operated-knee core score comprising three scored domains—Symptoms, Function, and Quality of Life—while the global postoperative Satisfaction item was retained as a separate appraisal.

Primary outcomes (reliability, validity, and responsiveness) were prespecified in the study protocol and guided the analyses reported here. TKA recovery involves physical, psychological, and social dimensions, and existing PROMs developed in Western contexts may omit activities and cultural concerns relevant to Chinese patients.^12^^,^^29^^,^^30^ Our qualitative work and item refinement emphasised culturally prevalent activities—such as stair climbing in buildings without elevators, squatting, and community exercise (Tai Chi, square dancing), thereby enhancing content validity for the target population.^15^^,^^30^, ^31^, ^32^, ^33^, ^34^, ^35^, ^36^, ^37^ The principal cultural contribution of the CTP lies in content relevance rather than cultural exclusivity of the underlying constructs. Its Symptoms and Function domains intentionally overlap with established instruments, whereas several activities and social-context items were retained because they were identified as relevant during target-population concept elicitation. Accordingly, the CTP should be viewed as complementary to, rather than a substitute for, established measures such as WOMAC.^31^

Methodologically, focussing analyses on the affected side reduced contamination from the contralateral limb and mitigated potential ceiling/floor effects.^32^^,^^33^ The question “Are you afraid of others seeing your knees and feeling embarrassed?” was removed from the “Quality of Life” dimension due to a floor effect The embarrassment item originated from patient concept elicitation during the previous qualitative development study rather than from direct adoption of an existing PROM. Although retained in the preliminary 35-item CTP, it showed a marked floor effect during quantitative refinement and was subsequently removed. We interpret this as evidence of poor item performance in the present study population rather than as evidence of a general cultural characteristic of Chinese patients.^37^, ^38^, ^39^, ^40^ This underscores the necessity of adapting PROMs to capture experiences relevant within specific sociocultural contexts.^41^ We therefore interpret its removal as an empirical item-performance finding in the present study population rather than as evidence of a general cultural characteristic of Chinese patients.

EFA supported a three-factor solution, whereas CFA provided mixed evidence: several absolute-fit indices met the prespecified criteria, but CFI and NNFI remained below the stricter thresholds. The proposed structure therefore received partial rather than uniformly strong support and requires independent replication. All three Expectation items showed pronounced ceiling concentrations and limited score dispersion; together with the other quantitative criteria and content review, these findings supported removal of the domain.^42^, ^43^, ^44^ Notably, the CTP enables assessment of bilateral knee pain, enhancing clinical relevance without increasing patient burden.^12^ The consolidated satisfaction item, while brief, retained utility for gauging overall patient appraisal and aligning care with patient expectations and adherence.^28^^,^^45^, ^46^, ^47^

The CTP showed high internal consistency and temporal stability (Cronbach's α = 0·864; ICC = 0·836), supporting score reliability in the study population. Convergent validity against WOMAC was supported by strong correlations between CTP Function and WOMAC Function and between CTP Symptoms and WOMAC Pain, consistent with the intended overlap between these constructs.^48^ The weak correlation between CTP Quality of Life and WOMAC Stiffness should be interpreted cautiously. WOMAC Stiffness is not a direct measure of quality of life, and the two domains were therefore not expected to show the same degree of convergence as the Function–Function and Symptoms–Pain comparisons. The weak association indicates limited construct overlap rather than providing strong validation of the CTP Quality-of-Life domain. Because no generic health-related quality-of-life or independent mental-health measure was collected, the convergent and discriminant validity of the CTP Quality-of-Life domain remains to be established in future studies.^49^^,^^50^

Responsiveness analyses showed that the CTP was sensitive to change at 1 year after TKA, with a total ES of 1.34 and an SRM of 1.66. Change was defined as the baseline score minus the 1-year score, such that positive values indicated improvement. WOMAC showed larger standardised changes for pain and physical function. However, effect-size magnitudes are influenced by an instrument's content, score distribution, and measurement target and should not be interpreted as a direct test of comparative superiority.^51^^,^^52^ The CTP also demonstrated moderate-to-large responsiveness across its Symptoms, Function, and Quality-of-Life domains, indicating that it can detect change in the outcomes represented by its item content. Its inclusion of target-population-relevant activities and social contexts provides complementary content coverage relative to WOMAC.15 Nevertheless, the present study did not evaluate whether the CTP adds explanatory or predictive information beyond WOMAC. Future studies should assess its incremental validity against independent external patient-centred outcomes. We therefore report WOMAC as a benchmark for comparison, while using the CTP to capture complementary information that may be particularly relevant for culturally tailored outcome assessment and for studies oriented toward patient-reported quality-of-life and social functioning.^53^, ^54^, ^55^, ^56^

Strengths of this study include a robust, mixed-methods development process grounded in a conceptual model specific to Chinese TKA patients, large multicenter samples across three validation stages, and the combined use of EFA and CFA to evaluate structural validity. We also assessed responsiveness—an essential property for longitudinal outcome measurement. The 16-item operated-knee core score demonstrated strong internal consistency and test–retest reliability, together with evidence of structural validity, convergent validity, and responsiveness.

Limitations merit mention. Although this was a multicentre study, all participating hospitals were tertiary centres in western China. The findings therefore establish multicentre evidence within this regional setting rather than nationwide external validity. Regional variation in socioeconomic conditions, health-care access, rehabilitation practices, language/dialect, occupations, and community activities may affect both item relevance and score distributions. Criterion validity could not be assessed owing to the absence of a recognised TKA-specific Chinese gold-standard PROM. The removal of expectation items—while methodologically justified—suggests that expectation-related constructs may require different measurement approaches or qualitative exploration. The CTP does not directly assess the specific construct of joint awareness or a “forgotten joint”. Instruments such as the Forgotten Joint Score therefore capture a complementary postoperative construct that is not represented explicitly in the CTP.

### Future research

Further work should focus on validation in geographically and socioeconomically diverse Chinese populations; evaluation of measurement invariance and differential item functioning; construct validation against generic HRQoL, mental-health, and joint-awareness measures; development of anchor-based interpretability thresholds; and assessment of predictive and incremental validity against external patient-centred outcomes.

### Conclusions

In conclusion, the CTP is a culturally grounded patient-reported outcome measure for Chinese patients undergoing TKA. Its 16-item operated-knee core score demonstrated satisfactory reliability, structural validity, convergent validity, and responsiveness. The CTP complements established measures such as WOMAC by incorporating target-population-relevant activities and social-context content. Future studies should establish anchor-based interpretability thresholds, evaluate incremental validity, and extend validation across diverse regions and health-care settings in China.

## Contributors

LS, JBM, and SXY contributed to the study conception and design. Material preparation, data collection and analysis were performed by CX, XFC, FY,HDW, GC, RZ. The first draft of the manuscript was written by CX, XFC and all authors commented on previous versions of the manuscript. All authors read and approved the final manuscript. Shuxin Yao, Lei Shang, and Jianbing Ma directly accessed and verified the underlying study data. Shuxin Yao, Lei Shang, and Jianbing Ma had final responsibility for the decision to submit the manuscript for publication.

## Data sharing statement

De-identified individual participant data underlying the results reported in this Article may be made available to qualified researchers beginning 6 months and ending 36 months after publication. Requests should be directed to the corresponding author and must include a scientifically appropriate research proposal. Data access will be subject to review and approval by the study investigators, compliance with applicable ethical and privacy requirements, and execution of a data-use agreement. The data will not be deposited in an unrestricted public repository because the participant consent and ethical approval for this study did not provide for unrestricted public release of individual-level clinical data. The data dictionary, study protocol, and analysis code will also be available on reasonable request. No accession code is applicable.

## Declaration of interests

All authors declare no competing interests.

## Appendix A Supplementary data

Translated abstract

Supplementary Material 1A

Supplementary Material 1B

Supplementary Methods S1

Supplementary Table S1A S1B
