# Supplementary material for: Quantitative refinement and multicentre psychometric validation of the Chinese total knee arthroplasty patient-reported outcome measure: a prospective cohort study with repeated measures
Source: Lancet Reg Health West Pac. 2026 Sep 19;74:101989. doi: 10.1016/j.lanwpc.2026.101989 (PMC13628658; doi:10.1016/j.lanwpc.2026.101989)
Supplement: Supplementary file 1 [file mmc1.docx]

This translation in Chinese was submitted by the authors, and we reproduce it as supplied. It has not been peer reviewed. Our editorial processes have only been applied to the original abstract in English, which should serve as reference for this manuscript.

**摘要**

**背景**： 全膝关节置换术（TKA）需要有效且具有文化适宜性的患者报告结局测量工具（PROMs）。此前，我们团队通过对中国患者开展定性研究并结合多学科专家意见，开发了一份包含35个条目的初步CTP，但该工具尚未经过定量精简或前瞻性多中心心理测量学验证。本研究旨在对该工具进行精简，并评价其术膝核心评分的心理测量学特性。

**方法**： 我们在西安、成都和兰州的三级医院开展了一项采用重复评估的前瞻性多中心队列心理测量学研究。参与者于2023年6月至2024年6月期间招募，12个月随访持续至2025年6月。采用三个独立队列分别进行定量条目筛选、最终量表构建和心理测量学评价。我们评估了内部一致性（Cronbach’s α）、重测信度（ICC）、结构效度、构念效度（与WOMAC的相关性）以及响应度（效应量[ES]；标准化反应均数[SRM]）。

**结果**： 三个分析队列分别纳入321、529和898名参与者（总计n=1748）。在三个队列中，平均年龄为66·5岁（SD 7·2），1195名参与者（68·4%）为女性。探索性因子分析支持三因子结构（功能、症状、生活质量），可解释72·2%的方差，所有条目的因子载荷均≥0·40。最终临床问卷包含22个条目。主要心理测量学分析评价了一个包含16个条目的术膝核心评分，该评分表现出较高的内部一致性（Cronbach’s α=0·864）和良好的重测信度（ICC=0·836）。其与WOMAC功能维度的强相关性支持了构念效度（r=0·698；95% CI 0·663–0·730；p<0·001）。在12个月时，术膝核心评分的平均值由33·52（SD 8·74）下降至21·81（SD 9·81），ES为1·34，SRM为1·66。

**解释**： 最终版CTP是一项包含22个条目、基于目标人群信息开发的临床PROM。其16条目的术膝核心评分表现出令人满意的信度和响应度，同时对所提出的三因子结构以及部分重叠构念的收敛效度提供了部分支持。

**资助**： 本研究获得国家自然科学基金（项目编号：82173627）、陕西省重点研发计划（编号：2023-YBSF-464）、西安市卫生健康委员会一般项目培育项目（编号：2024ms10）、西安市科技计划项目（25YXYJYB00002）以及陕西省创新团队项目（2023-CX-TD-73）的资助。
