# Supplementary material for: Quantitative refinement and multicentre psychometric validation of the Chinese total knee arthroplasty patient-reported outcome measure: a prospective cohort study with repeated measures
Source: Lancet Reg Health West Pac. 2026 Sep 19;74:101989. doi: 10.1016/j.lanwpc.2026.101989 (PMC13628658; doi:10.1016/j.lanwpc.2026.101989)
Supplement: Supplementary file 2 [file mmc2.docx]

**中国膝关节置换术患者报告结局测量表**
姓名：__________	日期：__________

请根据您在过去一个月内的膝关节状态，圈选最符合您感受的选项。

**1. 症状评价**

**1.1 双膝疼痛的严重程度及发生频率**

**左膝**

**疼痛严重程度**：○ 无 ○ 轻度 ○ 中度 ○ 严重 ○ 极严重

**疼痛发生频率**：○ 从不 ○ 很少 ○ 偶尔 ○ 经常 ○ 总是

**右膝**

**疼痛严重程度**：○ 无 ○ 轻度 ○ 中度 ○ 严重 ○ 极严重

**疼痛发生频率**：○ 从不 ○ 很少 ○ 偶尔 ○ 经常 ○ 总是

**1.2 双膝肿胀程度**

**左膝**：○ 无 ○ 轻度 ○ 中度 ○ 严重 ○ 极严重

**右膝**：○ 无 ○ 轻度 ○ 中度 ○ 严重 ○ 极严重

**1.3 双膝僵硬程度**

**左膝**：○ 无 ○ 轻度 ○ 中度 ○ 严重 ○ 极严重

**右膝**：○ 无 ○ 轻度 ○ 中度 ○ 严重 ○ 极严重

**1.4 双膝无力程度**

**左膝**：○ 无 ○ 轻度 ○ 中度 ○ 严重 ○ 极严重

**右膝**：○ 无 ○ 轻度 ○ 中度 ○ 严重 ○ 极严重

|  | 无困难 | 轻微困难 | 中等困难 | 严重困难 | 无法完成 |
| --- | --- | --- | --- | --- | --- |
| 平地行走 |  |  |  |  |  |
| 上下楼梯 |  |  |  |  |  |
| 下蹲 |  |  |  |  |  |
| 坐着穿脱袜子/鞋子 |  |  |  |  |  |
| 不借助扶手从低矮座位站起 |  |  |  |  |  |
| 轻度田间劳动（轻度农活） |  |  |  |  |  |
| 上厕所（坐下/起立） |  |  |  |  |  |
| 户外健身活动（太极、广场舞等） |  |  |  |  |  |

**2. 日常活动困难度**

**3. 心理与社交影响**

**3.1 您的膝关节状况对生活质量的影响程度**

○ 完全没有 ○ 略有影响 ○ 一定程度 ○ 中等程度 ○ 很大程度

**3.2 因膝痛影响，您外出拜访亲友的频率**

○ 从不 ○ 很少 ○ 偶尔 ○ 经常 ○ 总是

**3.3 您是否感到焦虑或抑郁**

○ 从不 ○ 很少 ○ 偶尔 ○ 经常 ○ 总是

**4. 手术满意度**

○ 非常不满意 ○ 不满意 ○ 一般 ○ 满意 ○ 非常满意

**评分说明**

请按下列方式给每题赋分，并计算总分：

**1.1 题（满分16分）**

疼痛严重程度：无 0；轻度 1；中度 2；严重 3；极严重 4

疼痛发生频率：从不 0；很少 1；偶尔 2；经常 3；总是 4

**1.2~1.4 题（各满分8分）**

肿胀／僵硬／无力程度：无 0；轻度 1；中度 2；严重 3；极严重 4

**2 题（满分32分）**

各活动困难度：无困难 0；轻微困难 1；中等困难 2；很大困难 3；无法完成 4

**3.1 题（满分4分）**

影响生活质量：完全没有 0；略有影响 1；一定程度 2；中等程度 3；很大程度 4

**3.2~3.3 题（各满分4分，共8分）**

频率/情绪：从不 0；很少 1；偶尔 2；经常 3；总是 4

**4 题（满分4分）**

手术满意度：非常不满意 0；不满意 1；一般 2；满意 3；非常满意 4

请将各部分得分相加，得出最终总分，以评估手术治疗效果。
