# Supplementary material for: Quantitative refinement and multicentre psychometric validation of the Chinese total knee arthroplasty patient-reported outcome measure: a prospective cohort study with repeated measures
Source: Lancet Reg Health West Pac. 2026 Sep 19;74:101989. doi: 10.1016/j.lanwpc.2026.101989 (PMC13628658; doi:10.1016/j.lanwpc.2026.101989)
Supplement: Supplementary file 3 [file mmc3.docx]

**Chinese Total Knee Arthroplasty Patient-Reported Outcome Measurement**

Name: Date:

Please circle the corresponding options based on your personal situation **within the past one month**

| 1.1 Please indicate the severity and frequency of pain in your knee joint. | | | | | | | | | | | | | | | | | | | | | | | |
| --- | --- | --- | --- | --- | --- | --- | --- | --- | --- | --- | --- | --- | --- | --- | --- | --- | --- | --- | --- | --- | --- | --- | --- |
| Left Knee | Pain | | Severity | | | None | | | | | Mild | | Moderate | | | | Severe | | | | | Very Severe | |
|  |  |  | Frequency | | | Never | | | | | Rarely | | Occasionally | | | | Frequently | | | | | Always | |
| Right Knee | Pain | | Severity | | | None | | | | Mild | | | Moderate | | | | Severe | | | | | Very Severe | |
|  |  |  | Frequency | | | Never | | | | Rarely | | | Occasionally | | | | Frequently | | | | | Always | |
| 1.2 Please indicate the severity of swelling in your knee joint. | | | | | | | | | | | | | | | | | | | | | | | |
| Right Knee | None | | | | Mild | | | | | | | Moderate | | | | Severe | | | | | Very Severe | | |
| Left Knee | None | | | | Mild | | | | | | | Moderate | | | | Severe | | | | | Very Severe | | |
| 1.3 Please indicate the severity of stiffness in your knee joint. | | | | | | | | | | | | | | | | | | | | | | | |
| Right Knee | None | | | | Mild | | | | | | | Moderate | | | | Severe | | | | | Very Severe | | |
| Left Knee | None | | | | Mild | | | | | | | Moderate | | | | Severe | | | | | Very Severe | | |
| 1.4 Please indicate the severity of weakness in your knee joint. | | | | | | | | | | | | | | | | | | | | | | | |
| Right Knee | None | | | | Mild | | | | | | | Moderate | | | | Severe | | | | | Very Severe | | |
| Left Knee | None | | | | Mild | | | | | | | Moderate | | | | Severe | | | | | Very Severe | | |
| 2. How difficult are you facing when doing the following activities? | | | | | | | | | | | | | | | | | | | | | | | |
|  | | | | | None | | | | Slight | | | | | Moderate | | | | Great | | | | | Unable |
| Walking on a flat surface | | | | |  | | | |  | | | | |  | | | |  | | | | |  |
| Climbing up or down stairs | | | | |  | | | |  | | | | |  | | | |  | | | | |  |
| Squatting | | | | |  | | | |  | | | | |  | | | |  | | | | |  |
| Taking off socks or shoes while sitting | | | | |  | | | |  | | | | |  | | | |  | | | | |  |
| Standing up from a low sitting position without using the armrests | | | | |  | | | |  | | | | |  | | | |  | | | | |  |
| Mild field labor | | | | |  | | | |  | | | | |  | | | |  | | | | |  |
| Getting on/off toilet | | | | |  | | | |  | | | | |  | | | |  | | | | |  |
| Outdoor fitness activities (Tai Chi or Square dancing) | | | | |  | | | |  | | | | |  | | | |  | | | | |  |
| 3.1 To what extent does your knee joint condition affect your quality of life? | | | | | | | | | | | | | | | | | | | | | | | |
| Not at all | | | | A little bit | | | | Somewhat | | | | | | A moderate amount | | | | | A lot | | | | |
| 3.2 How often does your affected knee prevent you from visiting friends and relatives? | | | | | | | | | | | | | | | | | | | | | | | |
| Never | | | | Rarely | | | | Occasionally | | | | | | Frequently | | | | | Always | | | | |
| 3.3 Do you feel anxious or depressed? | | | | | | | | | | | | | | | | | | | | | | | |
| Never | | | | Rarely | | | | Occasionally | | | | | | Frequently | | | | | Always | | | | |
| 4. Please rate your overall satisfaction with the surgical outcome. | | | | | | | | | | | | | | | | | | | | | | | |
| Very Dissatisfied | | Dissatisfied | | | | | Average | | | | | | | | Satisfied | | | | | Very Satisfied | | | |

**Chinese Total Knee Arthroplasty Patient-Reported Outcome Measurement**

**Scoring Instructions**

**Assign points as follows:**

**Question	Maximum Possible points**

**Question-1.1: 16**

Pain Severity: None = 0; Mild = 1; Moderate = 2; Severe = 3; Very Severe = 4.

Pain Frequency: Never = 0; Rarely = 1; Occasionally = 2; Frequently = 3; Always = 4.

**Question-1.2: 8**

Swelling Severity	None = 0; Mild = 1; Moderate = 2; Severe = 3; Very Severe = 4.

**Question-1.3: 8**

Stiffness Severity None = 0; Mild = 1; Moderate = 2; Severe = 3; Very Severe = 4.

**Question-1.4: 8**

Weakness Severity	None = 0; Mild = 1; Moderate = 2; Severe = 3; Very Severe = 4.

No Weakness = 0

**Question-2: 32**

All items	None = 0; Slight = 1; Moderate = 2; Great = 3; Unable = 4.

**Question-3.1: 4**

Affecting quality of life Not at all = 0; A little bit = 1; Somewhat = 2; A moderate amount = 3; A lot = 4.

**Question-3.2&3.3:** **8**

All items	Not at all = 0; A little bit = 1; Somewhat = 2; A moderate amount = 3; A lot = 4.

**Question-4:** **4**

Satisfaction	Very Dissatisfied = 0; Dissatisfied = 1; Average = 2; Satisfied = 3; Very Satisfied = 4.
