# Supplementary material for: Quantitative refinement and multicentre psychometric validation of the Chinese total knee arthroplasty patient-reported outcome measure: a prospective cohort study with repeated measures
Source: Lancet Reg Health West Pac. 2026 Sep 19;74:101989. doi: 10.1016/j.lanwpc.2026.101989 (PMC13628658; doi:10.1016/j.lanwpc.2026.101989)
Supplement: Supplementary file 4 [file mmc5.docx]

**Supplementary Table S1A. Item provenance and sequential refinement of the 35-item preliminary CTP to the final 22-item clinical questionnaire**

| **No.** | **Original domain** | **Original English item** | **Chinese item** | **Source/provenance** | **After Sample 1** | **After Sample 2 / final 22** | **Reason / final role** |
| --- | --- | --- | --- | --- | --- | --- | --- |
| 1 | Pain | Left-knee pain location | 左膝疼痛部位 | Literature-derived TKA PROM conceptual pool; wording refined through patient/expert elicitation and Delphi (Xu et al., 2025). | Removed | — | Removed in Sample 1 during prespecified multi-criterion quantitative screening; pain location was not retained in the later scoring structure. |
| 2 | Pain | Left-knee pain severity | 左膝疼痛严重程度 | Literature-derived conceptual pool; patient/expert refinement and Delphi (Xu et al., 2025). | Retained/reformatted | Retained | Final 22-item questionnaire; operated-knee response contributes to the 16-item core score. |
| 3 | Pain | Left-knee pain frequency | 左膝疼痛发生频率 | Literature-derived conceptual pool; patient/expert refinement and Delphi (Xu et al., 2025). | Retained/reformatted | Retained | Final 22-item questionnaire; operated-knee response contributes to the 16-item core score. |
| 4 | Pain | Right-knee pain location | 右膝疼痛部位 | Literature-derived TKA PROM conceptual pool; wording refined through patient/expert elicitation and Delphi (Xu et al., 2025). | Removed | — | Removed in Sample 1 during prespecified multi-criterion quantitative screening; pain location was not retained in the later scoring structure. |
| 5 | Pain | Right-knee pain severity | 右膝疼痛严重程度 | Literature-derived conceptual pool; patient/expert refinement and Delphi (Xu et al., 2025). | Retained/reformatted | Retained | Final 22-item questionnaire; operated-knee response contributes to the 16-item core score. |
| 6 | Pain | Right-knee pain frequency | 右膝疼痛发生频率 | Literature-derived conceptual pool; patient/expert refinement and Delphi (Xu et al., 2025). | Retained/reformatted | Retained | Final 22-item questionnaire; operated-knee response contributes to the 16-item core score. |
| 7 | Symptoms | Swelling location | 膝关节肿胀部位 | Literature-derived conceptual pool; patient/expert refinement and Delphi (Xu et al., 2025). | Retained/reformatted | Retained | The original location+severity format was restructured into separate left- and right-knee severity ratings; this was a format transformation, not an additional deletion. |
| 8 | Symptoms | Swelling severity | 膝关节肿胀严重程度 | Literature-derived conceptual pool; patient/expert refinement and Delphi (Xu et al., 2025). | Retained/reformatted | Retained | Reformatted as separate left- and right-knee severity ratings in the final questionnaire. |
| 9 | Symptoms | Stiffness location | 膝关节僵硬部位 | Literature-derived conceptual pool; patient/expert refinement and Delphi (Xu et al., 2025). | Retained/reformatted | Retained | The original location+severity format was restructured into separate left- and right-knee severity ratings; not counted as an additional deletion. |
| 10 | Symptoms | Stiffness severity | 膝关节僵硬严重程度 | Literature-derived conceptual pool; patient/expert refinement and Delphi (Xu et al., 2025). | Retained/reformatted | Retained | Reformatted as separate left- and right-knee severity ratings in the final questionnaire. |
| 11 | Symptoms | Weakness location | 膝关节无力部位 | Literature-derived conceptual pool; patient/expert refinement and Delphi (Xu et al., 2025). | Retained/reformatted | Retained | The original location+severity format was restructured into separate left- and right-knee severity ratings; not counted as an additional deletion. |
| 12 | Symptoms | Weakness severity | 膝关节无力严重程度 | Literature-derived conceptual pool; patient/expert refinement and Delphi (Xu et al., 2025). | Retained/reformatted | Retained | Reformatted as separate left- and right-knee severity ratings in the final questionnaire. |
| 13 | Function | Walking on a flat surface | 平地行走 | Literature-derived conceptual pool; patient/expert refinement and Delphi (Xu et al., 2025). | Retained | Retained | Final Function item; included in 16-item core score. |
| 14 | Function | Climbing up or down stairs | 上下楼梯 | Literature-derived conceptual pool; patient/expert refinement and Delphi (Xu et al., 2025). | Retained | Retained | Final Function item; included in 16-item core score. |
| 15 | Function | Squatting | 下蹲 | Literature-derived conceptual pool; patient/expert refinement and Delphi (Xu et al., 2025). | Retained | Retained | Final Function item; included in 16-item core score. |
| 16 | Function | Jogging | 慢跑 | Literature-derived conceptual pool; patient/expert refinement and Delphi (Xu et al., 2025). | Removed | — | Removed in Sample 1 after prespecified quantitative screening and clinical/content review. |
| 17 | Function | Taking off socks or shoes while sitting | 坐着穿脱袜子/鞋子 | Literature-derived conceptual pool; patient/expert refinement and Delphi (Xu et al., 2025). | Retained | Retained | Final Function item; included in 16-item core score. |
| 18 | Function | Standing from a low seat without arm support | 不借助扶手从低矮座位站起 | Literature-derived conceptual pool; patient/expert refinement and Delphi (Xu et al., 2025). | Retained | Retained | Final Function item; included in 16-item core score. |
| 19 | Function | Bath/Shower | 洗澡 | Literature-derived conceptual pool; patient/expert refinement and Delphi (Xu et al., 2025). | Removed | — | Removed in Sample 1 after prespecified quantitative screening and clinical/content review. |
| 20 | Function | Light household activity | 轻度家务活动（扫地、吸尘、清洁等） | Literature-derived/CHARLS-informed conceptual pool; patient/expert refinement and Delphi (Xu et al., 2025). | Removed | — | Removed in Sample 1 after prespecified quantitative screening and clinical/content review. |
| 21 | Function | Mild field labour | 轻度田间劳动（轻度农活） | Multisource conceptual pool with target-population refinement (Xu et al., 2025). | Retained | Retained | Final Function item; included in 16-item core score. |
| 22 | Function | Bending to floor | 弯腰捡拾地面物品 | Literature-derived conceptual pool; patient/expert refinement and Delphi (Xu et al., 2025). | Retained | Removed | Retained after Sample 1 but removed in Sample 2 during prespecified quantitative item reduction. |
| 23 | Function | Getting on/off toilet | 使用坐便器（坐下/起立） | Literature-derived conceptual pool; patient/expert refinement and Delphi (Xu et al., 2025). | Retained | Retained | Final Function item; included in 16-item core score. |
| 24 | Function | Getting in/out of a car | 进出小轿车 | Literature-derived conceptual pool; patient/expert refinement and Delphi (Xu et al., 2025). | Retained | Removed | Retained after Sample 1 but removed in Sample 2 during prespecified quantitative item reduction. |
| 25 | Function | Outdoor fitness activities (Tai Chi or Square dancing) | 户外健身活动（太极或广场舞） | Patient concept-elicitation interviews specifically added Tai Chi/Square dancing; refined through expert review/Delphi (Xu et al., 2025). | Retained | Retained | Final Function item; included in 16-item core score. |
| 26 | Quality of life | Impact on quality of life | 膝关节状况对生活质量的影响 | Literature-derived QoL concept pool; patient/expert refinement and Delphi (Xu et al., 2025). | Retained | Retained | Final QoL item; included in 16-item core score. |
| 27 | Quality of life | Impact on visiting friends and relatives | 患膝对探亲访友的影响 | Family-based social participation was prioritised during expert/patient qualitative refinement (Xu et al., 2025). | Retained | Retained | Final QoL item; included in 16-item core score. |
| 28 | Quality of life | Embarrassment when others see the knee | 担心他人看到膝关节而感到尴尬 | Present in the published preliminary CTP after the multisource qualitative/Delphi development process; no single external source was separately attributed (Xu et al., 2025). | Removed | — | Removed in Sample 1 because of a marked floor effect. |
| 29 | Quality of life | Anxious or depressed | 焦虑或抑郁 | Literature-derived mental/emotional concept pool, merged into QoL and refined through patient/expert input (Xu et al., 2025). | Retained | Retained | Final QoL item; included in 16-item core score. |
| 30 | Expectation | Expected pain relief | 期望疼痛缓解 | Expectation concept from the multisource TKA PROM conceptual pool; refined through patient/expert/Delphi development (Xu et al., 2025). | Removed | — | Removed in Sample 1 because of a marked ceiling effect. |
| 31 | Expectation | Expected recovery of activities of daily living | 期望恢复日常生活活动 | Expectation concept from the multisource TKA PROM conceptual pool; refined through patient/expert/Delphi development (Xu et al., 2025). | Removed | — | Removed in Sample 1 because of a marked ceiling effect. |
| 32 | Expectation | Expected recovery of leisure, recreational, or sport activities | 期望恢复休闲、娱乐或体育活动 | Expectation concept from the multisource TKA PROM conceptual pool; refined through patient/expert/Delphi development (Xu et al., 2025). | Removed | — | Removed in Sample 1 because of a marked ceiling effect. |
| 33 | Satisfaction | Satisfaction with pain relief | 对疼痛缓解的满意度 | Satisfaction concept from the multisource TKA PROM conceptual pool; refined through patient/expert/Delphi development (Xu et al., 2025). | Consolidated | Global item retained | Merged with the other two Satisfaction items after Sample 1 to reduce redundancy and retain a single global postoperative appraisal. |
| 34 | Satisfaction | Satisfaction with recovery of activities of daily living | 对日常生活活动恢复的满意度 | Satisfaction concept from the multisource TKA PROM conceptual pool; refined through patient/expert/Delphi development (Xu et al., 2025). | Consolidated | Global item retained | Merged with the other two Satisfaction items after Sample 1. |
| 35 | Satisfaction | Satisfaction with recovery of leisure/recreation/sport | 对休闲、娱乐或体育活动恢复的满意度 | Satisfaction concept from the multisource TKA PROM conceptual pool; refined through patient/expert/Delphi development (Xu et al., 2025). | Consolidated | Global item retained | Merged with the other two Satisfaction items after Sample 1. |
| D1 | Derived after Sample 1 | Global postoperative satisfaction | 总体术后满意度 | Derived by consolidation of the three original Satisfaction items after expert review in the present refinement study. | New consolidated item | Retained | Final 22-item clinical questionnaire; scored 0–4 and reported separately from the 16-item core psychometric score. |

**Notes:**

1. The preliminary 35-item CTP was developed previously through a targeted review of TKA-related PROMs and relevant content from the China Health and Retirement Longitudinal Study (CHARLS), concept elicitation with patients and multidisciplinary experts, two rounds of Delphi consultation, and cognitive debriefing (Xu C, Wei J, Li L, et al. Quality of Life Research. 2025;34:763–775). Because the previous study did not assign a single unique source to each item, provenance is reported by developmental source category rather than by unsupported item-specific attribution.

2. For swelling, stiffness, and weakness, the original location-plus-severity response structure was reformatted during quantitative refinement into separate left- and right-knee severity ratings; this was treated as a format restructuring rather than as additional item deletion.

3. After Sample 1, nine original items were removed (two pain-location items, three Function items, one Quality-of-Life item, and all three Expectation items). The three original Satisfaction items were then consolidated into one global postoperative Satisfaction item, yielding the 24-item trial questionnaire administered in Sample 2.

4. Sample 2 removed two further Function items (Bending to floor and Getting in/out of a car), yielding the final 22-item clinical questionnaire: 10 bilateral symptom items, eight Function items, three Quality-of-Life items, and one global Satisfaction item.

5. For psychometric evaluation, the operated-knee core score includes five operated-knee Symptoms items, eight Function items, and three Quality-of-Life items (16 items; total score 0–64). The five contralateral-knee symptom items are retained for bilateral clinical description, and the global Satisfaction item is a separate postoperative appraisal (0–4); neither is included in the core latent score.

6. Phase 1 item-level descriptive statistics for the analysed response variables are provided in Supplementary Table S1B.

**Supplementary Table S1B. Phase 1 descriptive statistics for analysed response variables corresponding to the original 35-item CTP pool (n=321)**

| **No.** | **Domain** | **Original item** | **Response format** | **N** | **Mean** | **SD** | **Skewness** | **Floor, n (%)** | **Ceiling, n (%)** | **Stage-specific disposition** |
| --- | --- | --- | --- | --- | --- | --- | --- | --- | --- | --- |
| 1 | Pain | Left knee pain location† | Categorical/multiple response | 321 | — | — | — | — | — | Removed—Phase 1 |
| 2 | Pain | Left knee pain severity | Ordinal (0–4) | 321 | 2.20 | 1.30 | −0.43 | 49 (15.3) | 43 (13.4) | Retained after Phase 1 |
| 3 | Pain | Left knee pain frequency | Ordinal (0–4) | 321 | 2.36 | 1.22 | −0.87 | 49 (15.3) | 35 (10.9) | Retained after Phase 1 |
| 4 | Pain | Right knee pain location† | Categorical/multiple response | 321 | — | — | — | — | — | Removed—Phase 1 |
| 5 | Pain | Right knee pain severity | Ordinal (0–4) | 321 | 2.00 | 1.16 | −0.59 | 59 (18.4) | 10 (3.1) | Retained after Phase 1 |
| 6 | Pain | Right knee pain frequency | Ordinal (0–4) | 321 | 2.19 | 1.19 | −0.85 | 59 (18.4) | 16 (5.0) | Retained after Phase 1 |
| 7 | Symptoms | Left knee swelling severity§ | Ordinal (0–4) | 321 | 0.84 | 1.12 | 0.90 | 187 (58.3) | 0 (0.0) | Retained after Phase 1 |
| 8 | Symptoms | Right knee swelling severity§ | Ordinal (0–4) | 321 | 0.75 | 1.02 | 1.03 | 186 (57.9) | 0 (0.0) | Retained after Phase 1 |
| 9 | Symptoms | Left knee stiffness severity§ | Ordinal (0–4) | 321 | 1.69 | 1.41 | −0.05 | 111 (34.6) | 19 (5.9) | Retained after Phase 1 |
| 10 | Symptoms | Right knee stiffness severity§ | Ordinal (0–4) | 321 | 1.31 | 1.29 | 0.32 | 134 (41.7) | 5 (1.6) | Retained after Phase 1 |
| 11 | Symptoms | Left knee weakness severity§ | Ordinal (0–4) | 321 | 1.56 | 1.20 | −0.09 | 95 (29.6) | 5 (1.6) | Retained after Phase 1 |
| 12 | Symptoms | Right knee weakness severity§ | Ordinal (0–4) | 321 | 1.34 | 1.19 | 0.09 | 122 (38.0) | 0 (0.0) | Retained after Phase 1 |
| 13 | Function | Walking on a flat surface | Ordinal (0–4) | 321 | 1.73 | 1.10 | −0.04 | 49 (15.3) | 8 (2.5) | Retained after Phase 1 |
| 14 | Function | Climbing up or down stairs | Ordinal (0–4) | 321 | 2.75 | 0.77 | −0.31 | 0 (0.0) | 46 (14.3) | Retained after Phase 1 |
| 15 | Function | Squatting | Ordinal (0–4) | 321 | 3.13 | 1.15 | −1.41 | 22 (6.9) | 163 (50.8) | Retained after Phase 1 |
| 16 | Function | Jogging | Ordinal (0–4) | 321 | 3.62 | 0.66 | −1.48 | 0 (0.0) | 230 (71.7) | Removed—Phase 1 |
| 17 | Function | Taking off socks or shoes while sitting | Ordinal (0–4) | 321 | 0.67 | 1.00 | 1.39 | 201 (62.6) | 8 (2.5) | Retained after Phase 1 |
| 18 | Function | Standing from a low seat without arm support | Ordinal (0–4) | 321 | 1.45 | 1.34 | 0.47 | 105 (32.7) | 27 (8.4) | Retained after Phase 1 |
| 19 | Function | Bath/Shower | Ordinal (0–4) | 321 | 0.86 | 0.97 | 0.86 | 147 (45.8) | 0 (0.0) | Removed—Phase 1 |
| 20 | Function | Light household activity | Ordinal (0–4) | 321 | 1.07 | 0.96 | 0.99 | 94 (29.3) | 10 (3.1) | Removed—Phase 1 |
| 21 | Function | Mild field labour | Ordinal (0–4) | 321 | 2.60 | 1.08 | −0.79 | 19 (5.9) | 58 (18.1) | Retained after Phase 1 |
| 22 | Function | Bending to floor | Ordinal (0–4) | 321 | 1.19 | 1.11 | 0.63 | 109 (34.0) | 9 (2.8) | Retained after Phase 1; removed—Phase 2 |
| 23 | Function | Getting on/off toilet | Ordinal (0–4) | 321 | 1.04 | 1.12 | 0.78 | 139 (43.3) | 8 (2.5) | Retained after Phase 1 |
| 24 | Function | Getting in/out of a car | Ordinal (0–4) | 321 | 1.90 | 1.00 | −0.44 | 34 (10.6) | 0 (0.0) | Retained after Phase 1; removed—Phase 2 |
| 25 | Function | Outdoor fitness activities | Ordinal (0–4) | 321 | 2.60 | 1.21 | −0.40 | 14 (4.4) | 97 (30.2) | Retained after Phase 1 |
| 26 | Quality of life | Impact on quality of life | Ordinal (0–4) | 321 | 3.36 | 0.75 | −1.15 | 0 (0.0) | 160 (49.8) | Retained after Phase 1 |
| 27 | Quality of life | Impact on visiting friends and relatives | Ordinal (0–4) | 321 | 2.56 | 1.03 | −0.97 | 21 (6.5) | 40 (12.5) | Retained after Phase 1 |
| 28 | Quality of life | Embarrassment when others see the knee | Ordinal (0–4) | 321 | 1.02 | 1.16 | 1.03 | 138 (43.0) | 14 (4.4) | Removed—Phase 1 |
| 29 | Quality of life | Anxious or depressed | Ordinal (0–4) | 321 | 2.11 | 1.32 | −0.32 | 60 (18.7) | 45 (14.0) | Retained after Phase 1 |
| 30 | Expectation | Expected pain relief | Ordinal (0–4) | 321 | 3.95 | 0.22 | −4.01 | 0 (0.0) | 304 (94.7) | Removed—Phase 1 |
| 31 | Expectation | Expected recovery of activities of daily living | Ordinal (0–4) | 321 | 3.82 | 0.44 | −2.52 | 0 (0.0) | 272 (84.7) | Removed—Phase 1 |
| 32 | Expectation | Expected recovery of leisure/recreation/sport | Ordinal (0–4) | 321 | 3.35 | 0.93 | −1.69 | 10 (3.1) | 182 (56.7) | Removed—Phase 1 |
| 33 | Satisfaction | Satisfaction with pain relief‡ | Not administered | — | — | — | — | — | — | Consolidated into one global item after Phase 1 |
| 34 | Satisfaction | Satisfaction with recovery of activities of daily living‡ | Not administered | — | — | — | — | — | — | Consolidated into one global item after Phase 1 |
| 35 | Satisfaction | Satisfaction with recovery of leisure/recreation/sport‡ | Not administered | — | — | — | — | — | — | Consolidated into one global item after Phase 1 |

**Notes:** CTP=Chinese Total Knee Arthroplasty Patient-Reported Outcome Measurement. Ordered response categories were coded from 0 (lowest) to 4 (highest). Floor and ceiling percentages are the proportions selecting categories 0 and 4, respectively. A proportion >15% was treated as one prespecified item-performance flag, not as an automatic deletion rule; final decisions were based on the combined quantitative criteria and clinical/content review. Values are shown as n (%) where applicable.

† Pain-location items used a categorical/multiple-response format; ordinal mean, SD, skewness, and floor/ceiling statistics were not applicable. Left knee: no pain 49 (15.3%), one location 140 (43.6%), multiple locations 132 (41.1%). Right knee: no pain 59 (18.4%), one location 164 (51.1%), multiple locations 98 (30.5%).

‡ The three Satisfaction items referred to postoperative appraisal and were not administered as true responses at the preoperative Phase 1 screening time point. Database placeholder zeros were excluded from descriptive statistics; the three items were consolidated into one global Satisfaction item after Sample 1.

§ For swelling, stiffness, and weakness, the Phase 1 analytic dataset stored separate left- and right-knee severity ratings following the format restructuring described in Supplementary Table S1A. Accordingly, rows 7–12 report the six analysed response variables corresponding collectively to original items 7–12, rather than a literal one-to-one reproduction of the original location-plus-severity wording.

The sequential item provenance and refinement pathway (35→26→24→22) is shown in Supplementary Table S1A.
