# Supplementary material for: Quantitative refinement and multicentre psychometric validation of the Chinese total knee arthroplasty patient-reported outcome measure: a prospective cohort study with repeated measures
Source: Lancet Reg Health West Pac. 2026 Sep 19;74:101989. doi: 10.1016/j.lanwpc.2026.101989 (PMC13628658; doi:10.1016/j.lanwpc.2026.101989)
Supplement: Supplementary file 5 [file mmc4.doc]

PubMed

((("Patient Outcome Assessment"[Mesh]) OR ((("patient" or "self") AND ("report" or "reported" or "reporting" or "rated" or "rating" or "based" or "assessed")) AND ("measure" or "measurement" or "scale" or "instrument" or "questionnaire"))) AND ((((((("structural validity" OR "factor analysis" OR "factor analyses") OR (("internal consistency") OR (cronbach AND (alpha OR alphas)))) OR ((cross-cultural or "cross cultural") AND (equivalence or validity))) OR ((test-retest OR (test AND retest)) AND (reliab*))) OR ((("measurement error") OR (((smallest OR minimal OR minimally OR clinical OR clinically) AND (important OR significant OR detectable)) AND (change OR difference))) OR ("limits of agreement"))) OR ("construct validity" OR concordance OR discriminative OR "known group")) OR (reproducib* or responsive*))) AND ((knee) AND (arthroplasty or prosthesis or replacement or resurfacing))

Embase

('patient-reported outcome'/exp OR 'patient-reported outcome' OR (('patient' OR 'self') AND ('report' OR 'reported' OR 'reporting' OR 'rated' OR 'rating' OR 'based' OR 'assessed') AND ('measure' OR 'measurement' OR 'scale' OR 'instrument' OR 'questionnaire'))) AND ('structural validity' OR 'factor analysis' OR 'factor analyses' OR 'internal consistency' OR (cronbach AND (alpha OR alphas)) OR ('cross cultural' AND (equivalence OR validity)) OR (('test retest' OR (test AND retest)) AND reliab*) OR 'measurement error' OR ((smallest OR minimal OR minimally OR clinical OR clinically) AND (important OR significant OR detectable) AND (change OR difference)) OR 'limits of agreement' OR 'construct validity' OR concordance OR discriminative OR 'known group' OR reproducib* OR responsive*) AND knee AND (arthroplasty OR prosthesis OR replacement OR resurfacing)

Scopus

( ( ( TITLE-ABS ( "patient" OR "self" ) ) AND ( TITLE-ABS ( "report" OR "reported" OR "reporting" OR "rated" OR "rating" OR "based" OR "assessed" ) ) AND ( TITLE-ABS ( "measure" OR "measurement" OR "scale" OR "instrument" OR "questionnaire" ) ) ) OR ( TITLE-ABS ( "Patient Outcome Assessment" ) ) ) AND ( ( ( TITLE-ABS ( "smallest" OR "minimal" OR "minimally" OR "clinical" OR "clinically" ) ) AND ( TITLE-ABS ( "important" OR "significant" OR "detectable" ) ) AND ( TITLE-ABS ( "change" OR "difference" ) ) ) OR ( ( TITLE-ABS ( "test retest" ) ) AND ( TITLE-ABS ( "reliab*" ) ) ) OR ( ( TITLE-ABS ( "cross cultural" ) ) AND ( TITLE-ABS ( "equivalence" OR "validity" ) ) ) OR ( TITLE-ABS ( "structural validity" OR "factor analysis" OR "factor analyses" ) ) OR ( TITLE-ABS ( "internal consistency" ) ) OR ( TITLE-ABS ( "cronbach alpha" ) ) OR ( TITLE-ABS ( "measurement error" ) ) OR ( TITLE-ABS ( "limits of agreement" ) ) OR ( TITLE-ABS ( "construct validity" OR "concordance" OR "discriminative" OR "known group" ) ) OR ( TITLE-ABS ( "reproducib*" OR "responsive*" ) ) ) AND ( ( TITLE-ABS ( "arthroplasty" OR "prosthesis" OR "replacement" OR "resurfacing" ) ) AND ( knee ) )
